# An unusual site of ablation for a ventricular tachycardia

**DOI:** 10.1002/joa3.12954

**Published:** 2023-11-14

**Authors:** Anindya Ghosh, Anbarasan Sekar, Sabari Saravanan, Chenni S. Sriram, Ulhas M. Pandurangi

**Affiliations:** ^1^ Department of Cardiac Electrophysiology and Pacing, Arrhythmia Heart Failure Academy The Madras Medical Mission Chennai Tamil Nadu India; ^2^ Abbott EP, India Chennai Tamil Nadu India; ^3^ Division of Cardiology Sub‐section of Electrophysiology, Children's Hospital of Michigan and Detroit Medical Center Detroit Michigan USA

**Keywords:** Amplatzer post‐MI VSD occluder, implantable prosthetic device, isthmus over occluder device, ventricular septal rupture, ventricular tachycardia ablation

## Abstract

Scar‐related ventricular tachycardia (VT) ablation involves localizing the critical isthmuses by overdrive pacing maneuvers and three‐dimensional activation mapping. Implantable prosthetic devices have been known to complicate this by covering sites of potential isthmuses. We herein present a sentinel report of scar‐VT ablation with a protected isthmus localized over an endothelialized post‐myocardial infarction ventricular septal defect occluder device.

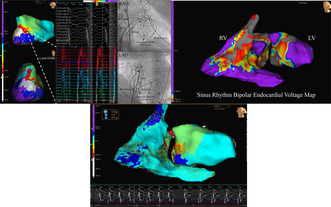

A 62‐year‐old male with congestive heart failure (CHF, NYHA class III symptoms) secondary to ischemic cardiomyopathy (ejection fraction of 25%) presented with incessant palpitations for a duration of one‐week. He had incurred an inferior wall myocardial infarction 16 years ago complicated by rupture of the basal ventricular septum. This was treated by coronary artery bypass grafting for double vessel disease involving the right coronary and left anterior descending arteries along with ventricular septal defect (VSD) patch closure. Two years ago, he underwent percutaneous closure of a residual VSD patch defect with a 20 mm Amplatzer™ post‐infarct muscular VSD occluder. This was done to ameliorate refractory CHF symptoms despite guideline‐directed medical therapy.

His presenting 12‐lead electrocardiogram (ECG) revealed a regular monomorphic wide QRS tachycardia (rate 160 bpm) which was diagnosed as ventricular tachycardia (VT, Figure 1) for reasons enunciated below. There was an atypical left bundle branch block (LBBB) morphology in lead V1 with initial instrinsicoid deflection >60 ms, QRS duration of 150 ms and late precordial transition by lead V4. The mean frontal QRS axis was 20 degrees. Atrio‐ventricular (AV) dissociation could not be ruled out given the variability/undulating morphology of the T waves. Most importantly, his baseline 12‐lead ECG during sinus rhythm (Figure 1B) demonstrated a typical right bundle branch block morphology (albeit with superimposed post‐infarct changes) and a wider QRS duration (200 ms) than tachycardia.^1^, ^2^ A preliminary review of the morphology of the VT is suggestive of a basal septal origin/exit depending on focal or reentrant mechanism, respectively.

**FIGURE 1:**
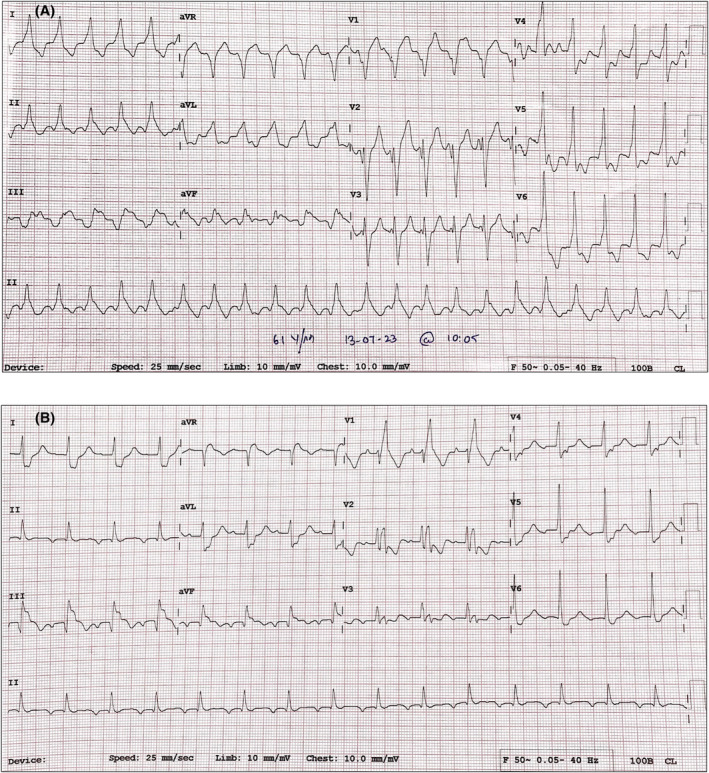
(A) 12‐lead electrocardiogram of the left bundle branch block (LBBB) type morphology tachycardia with QRS duration of 150 milliseconds (ms) (See text for description). (B) Baseline 12‐lead ECG showing sinus rhythm, normal PR interval and right bundle branch block with QRS duration of 200 milliseconds (ms). Note the prominent notching of the QRS complex in V2‐V3 suggesting septal fibrosis.

He underwent an invasive electrophysiology procedure. A sinus rhythm voltage with low voltage areas in the basal septum is available for both ventricles in Figure S1 and for LV in Figure S2.The hemodynamically stable clinical VT (cycle length or CL of 480 ms) was easily and reproducibly induced with atrial overdrive pacing. Endocardial activation mapping (Abbott Ensite Precision™) of the VT was performed using a multipolar HD grid catheter in the right as well as the left ventricle (retrograde aortic approach). The peak intrinsicoid QRS deflection in lead V5 during VT was used as the fiducial reference for mapping. Window settings were calibrated to represent 90% of the CL (−263 ms to +176 ms). The activation map was consistent with a basal left ventricular macro‐reentrant VT. The entire CL was accounted for along with an ‘early meets late’ isochronal activation pattern. There was isochronal color crowding in a relatively narrow zone in the basal mid‐septum suggestive of decremental conduction. Concurrent ventricular electrograms (EGMs) at this site demonstrated prolonged fractionated near‐field mid to late diastolic signals representing 70% of the CL. The above findings are illustrated and labelled in Figure 2A. A schema of VT circuit is explicated in Figure S3, and a biventricular VT propagation map is shown in Video S1.

**FIGURE 2:**
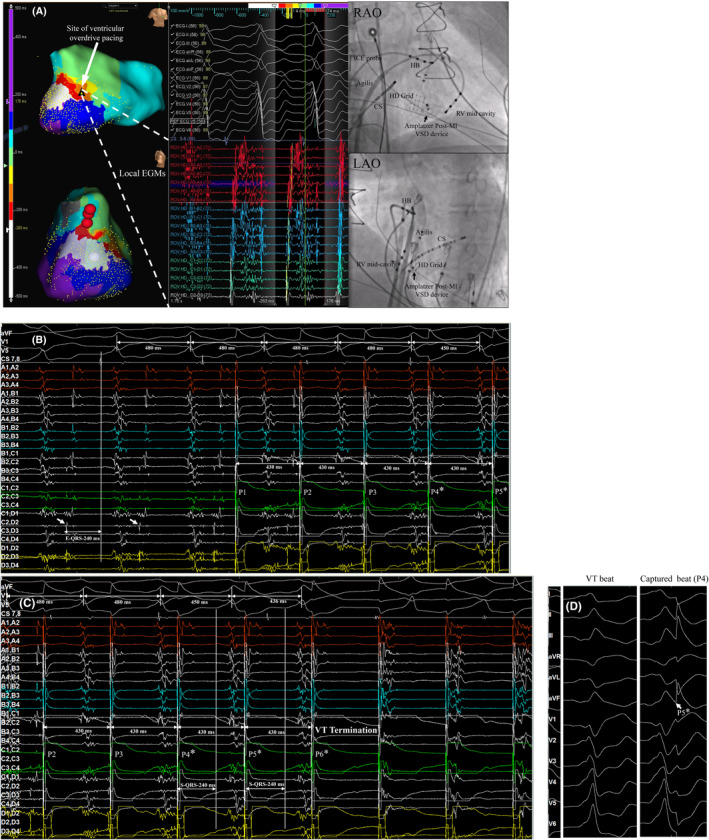
(A) Mapping left ventricular endocardium during tachycardia. Left panel shows three‐dimensional isochronal activation map during VT {Right anterior oblique (RAO) and Right lateral projections}. Red balls indicate location of his bundle potential. The entire cycle length (CL) was accounted for along with an ‘early meets late’ isochronal activation pattern. There was isochronal color crowding in a relatively narrow zone in the basal mid‐septum suggestive of decremental conduction/isthmus. Middle panel shows concurrent near‐field multicomponent fractionated electrograms in the basal mid septum in mid‐diastole encompassing nearly 70% of tachycardia CL. Right panel shows orthogonal fluoroscopic projections (RAO and left anterior oblique) with labelled hardware and catheter positions. Note the presence of VSD occlude device in basal mid septum. The multipolar HD Grid catheter sits over the device recording near‐field fractionated mid‐diastolic signals and isochronal color crowding. (B) Intra‐cardiac recordings from multipolar HD grid catheter over the VSD occlude device. EGM to QRS from C2D2 bipolar electrode is 240 ms. This is followed by overdrive pacing from same set of electrodes at 430 ms (See the text for details). (C) Intra‐cardiac recordings (continuation of the trace from (B)) showing concealed fusion with reset and tachycardia termination (see the text for details). (D) 12‐lead ECG morphology comparison of VT and pacing capture with concealed fusion (see the text for details). Partial distortion of the entrained beat by the subsequent pacing stimulus is also seen.

Overdrive pacing was performed (50 ms faster than the tachycardia CL) at a site with fractionated EGMs (late‐systole to mid‐diastole) that was adjacent to the area of isochronal color crowding (Figure 2B,C). Pacing stimuli were delivered from the C2, D2 bipole of multipolar HD grid catheter. Near‐field capture of the mid‐diastolic EGMs during VT is only seen from 4th‐6th pacing stimuli (annotated by * in Figure 2B,C). There is immediate reset of the VT following the first captured beat (P4) with similar QRS morphology, albeit with some distortion due to pacing artifact (Figure 2C,D). The near‐field EGM to QRS during VT (Figure 2B) was prolonged (240 ms) and identical to pacing stimulus to the captured QRS (Figure 2C). The last paced/captured beat (P6) during VT also terminates it. This is followed by paced QRS beats.

The interpretation here is that there is concealed fusion with reset (concealed entrainment) of the reentrant VT while pacing from a protected isthmus (S‐QRS = E‐QRS = 50% of TCL). In this context, the termination of VT is also a form of reset due to exit block.^3^ The fact that we could re‐induce the same VT and finally document its termination during subsequent radiofrequency ablation (RFA) at the same site is additional validation of the above (Figure 3A). The RFA settings and characteristics are shown in Figure S4.

**FIGURE 3:**
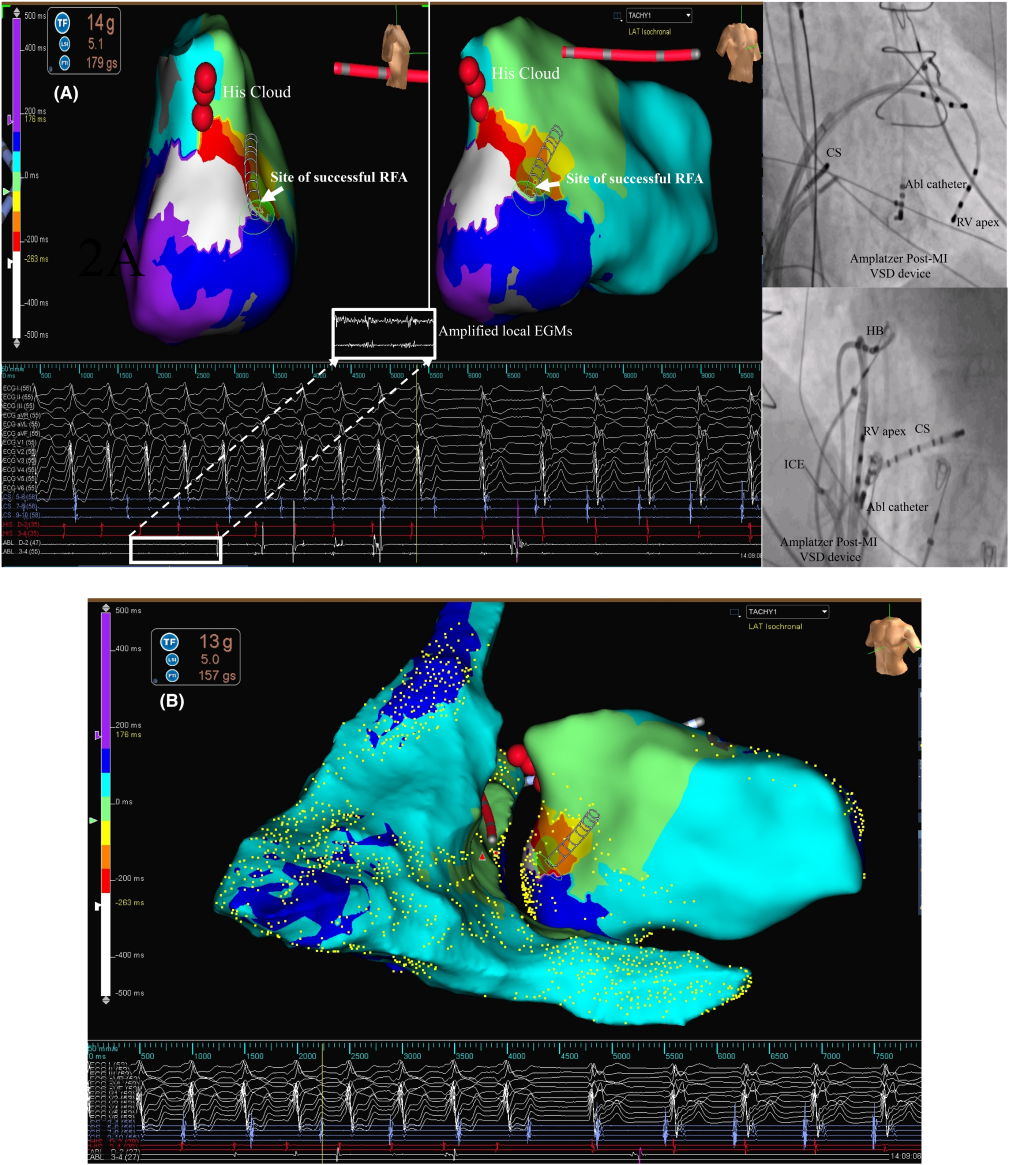
(A) Radiofrequency ablation during VT. 3‐dimentional activation map of LV during VT during ablation at the target site (Figure 2A). The tachycardia terminated with resumption of sinus rhythm. Right panel shows orthogonal fluoroscopic projections (RAO and left anterior oblique) with labelled hardware and catheter positions. Note the quadripolar contact‐force ablation catheter retrogradely placed over the occluder device at the point of tachycardia termination. (B) Biventricular endocardial three‐dimensional activation map of the VT with ablation catheter at the site of successful ablation. Note the entire VT CL is accommodated in the left ventricular basal endocardial aspect. Right ventricular activation is passive.

In our case, the site of postulated VT isthmus (measuring 11 mm in width as illustrated in the three‐dimensional map in Figure S3)/successful ablation correlated fluoroscopically with the area on and around the left ventricular endocardial aspect of the 20 mm VSD septal occluder (Figures 2A and 3A). Its relationship to the ventricular septum can be readily conjectured from the three‐dimensional electro‐anatomical map of both the ventricles (Figure 3B).

Endothelialization of occluder devices used for atrial septal defects (ASD) typically occurs within 6 months to 2 years.^4^ This experience is likely extrapolated to VSD septal occluder devices as well. This neo‐hyperplastic tissue is typically devoid of myocardial cells and is traditionally conceptualized as a non‐conducting scar. However, there are reports of myofibroblast cell populations in neo‐endothelialized ASD devices.^5^ Myofibroblasts are an integral component within myocardial infarct scars. They represent a likely substrate for slow conduction when electrically coupled with surviving myocardial tissue arranged in a zig‐zag fashion.^6^, ^7^

Despite all the presented supporting evidence, there was no robust histopathological evidence to confirm the presence of regenerated myocardium on the device and peri‐device location of the VT isthmus still remains a possibility.

In this context, a zone of slow conduction responsible for a VT isthmus/site of successful ablation on the endocardial aspect of a potentially endothelialized VSD septal occluder device is hypothesis generating. To the best of our knowledge, this represents a sentinel report.

## CONFLICT OF INTEREST STATEMENT

Authors declare no conflict of interests for this article.

## Supporting information


Data S1.

## Data Availability

The data that support the findings of this study are available from the corresponding author upon reasonable request.
