# Supplementary material for: An unusual site of ablation for a ventricular tachycardia
Source: J Arrhythm. 2023 Nov 14;40(1):160–5. doi: 10.1002/joa3.12954 (PMC10848586; doi:10.1002/joa3.12954)
Supplement: Supplementary file 1 — Data S1. [file JOA3-40-160-s001.zip › joa312954-sup-0001-Figures.pptx]

## Slide 1
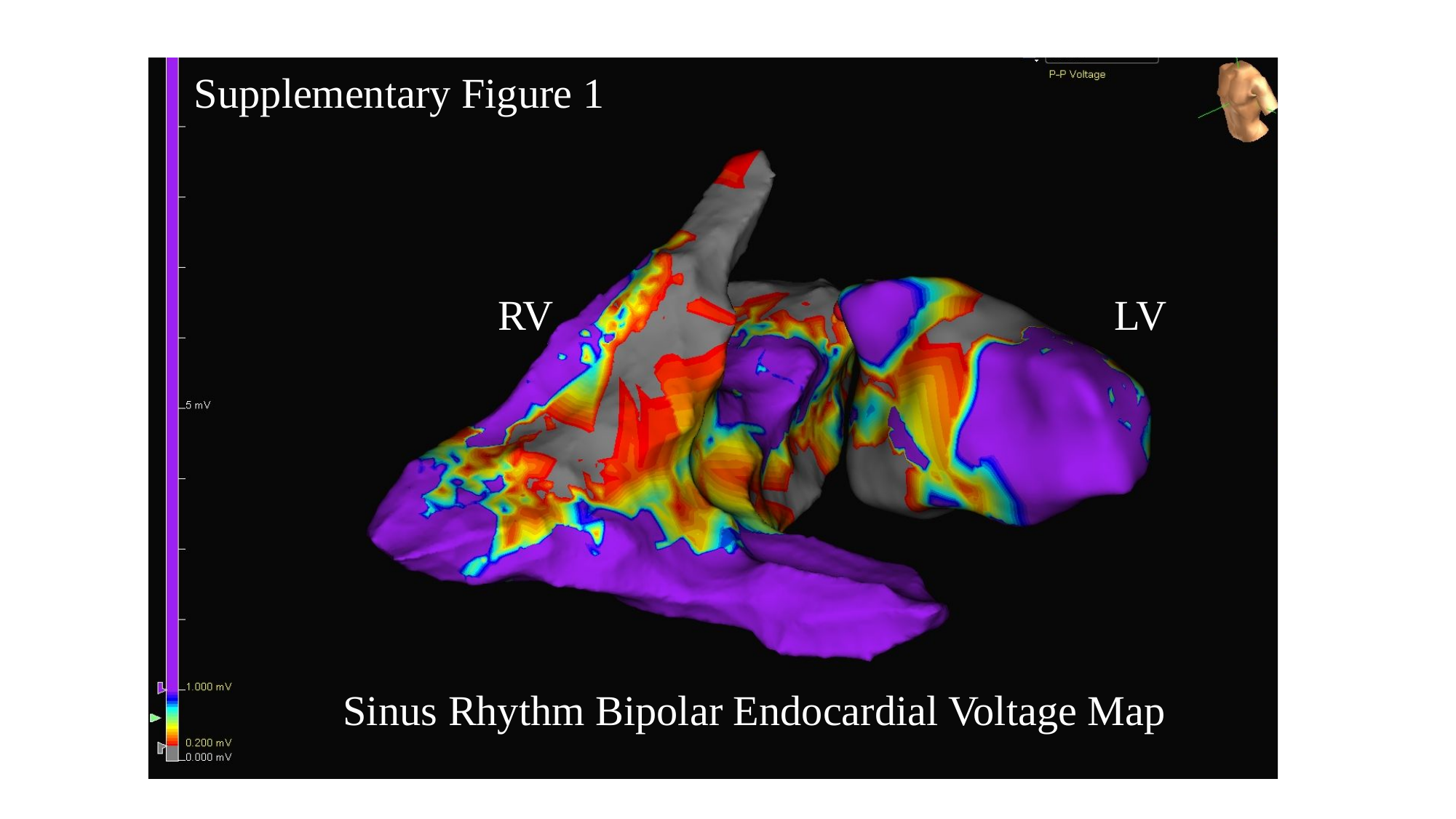

Supplementary Figure 1
LV
RV
Sinus Rhythm Bipolar Endocardial Voltage Map

## Slide 2
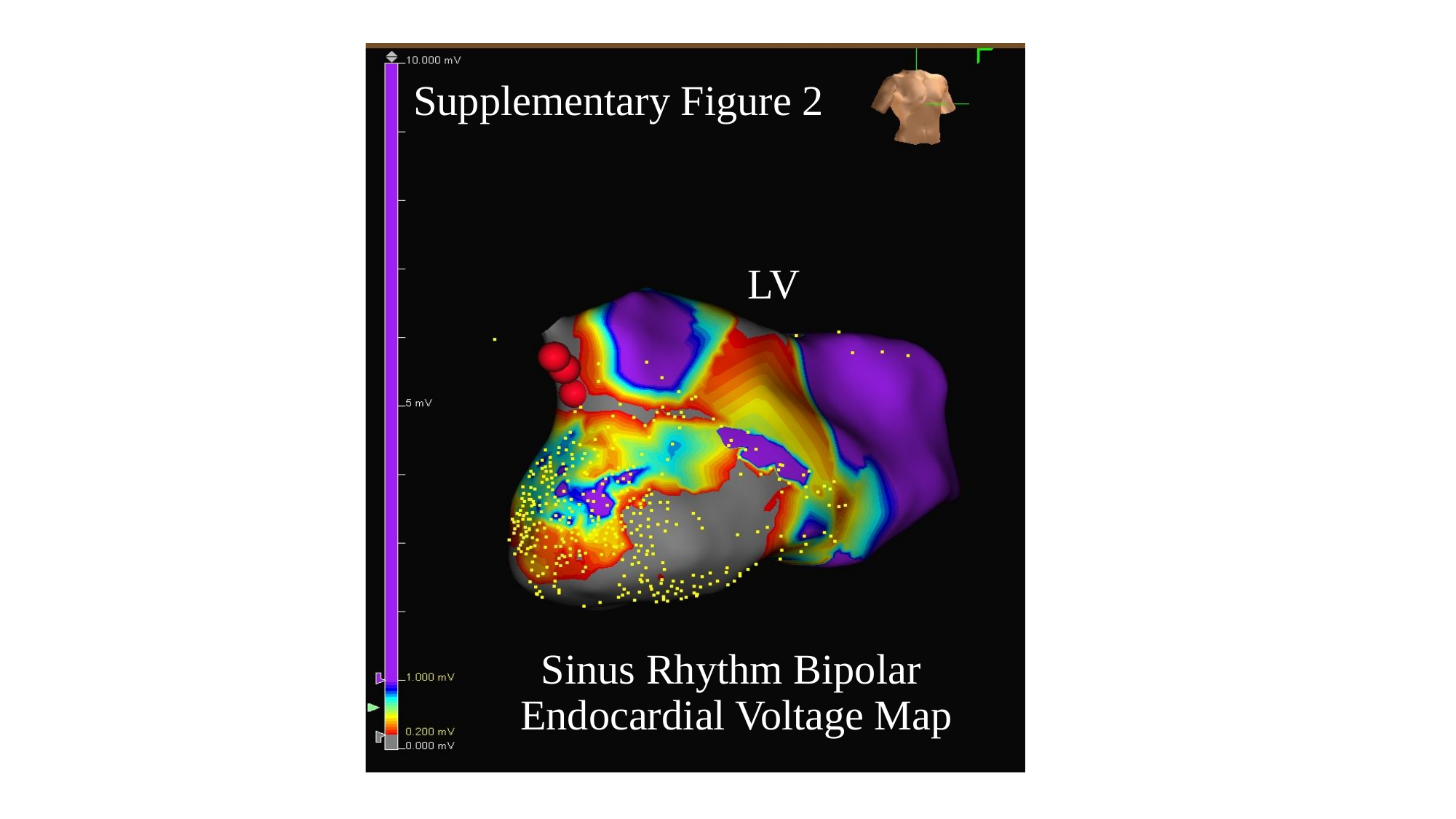

Supplementary Figure 2
LV
Sinus Rhythm Bipolar
Endocardial Voltage Map

## Slide 3
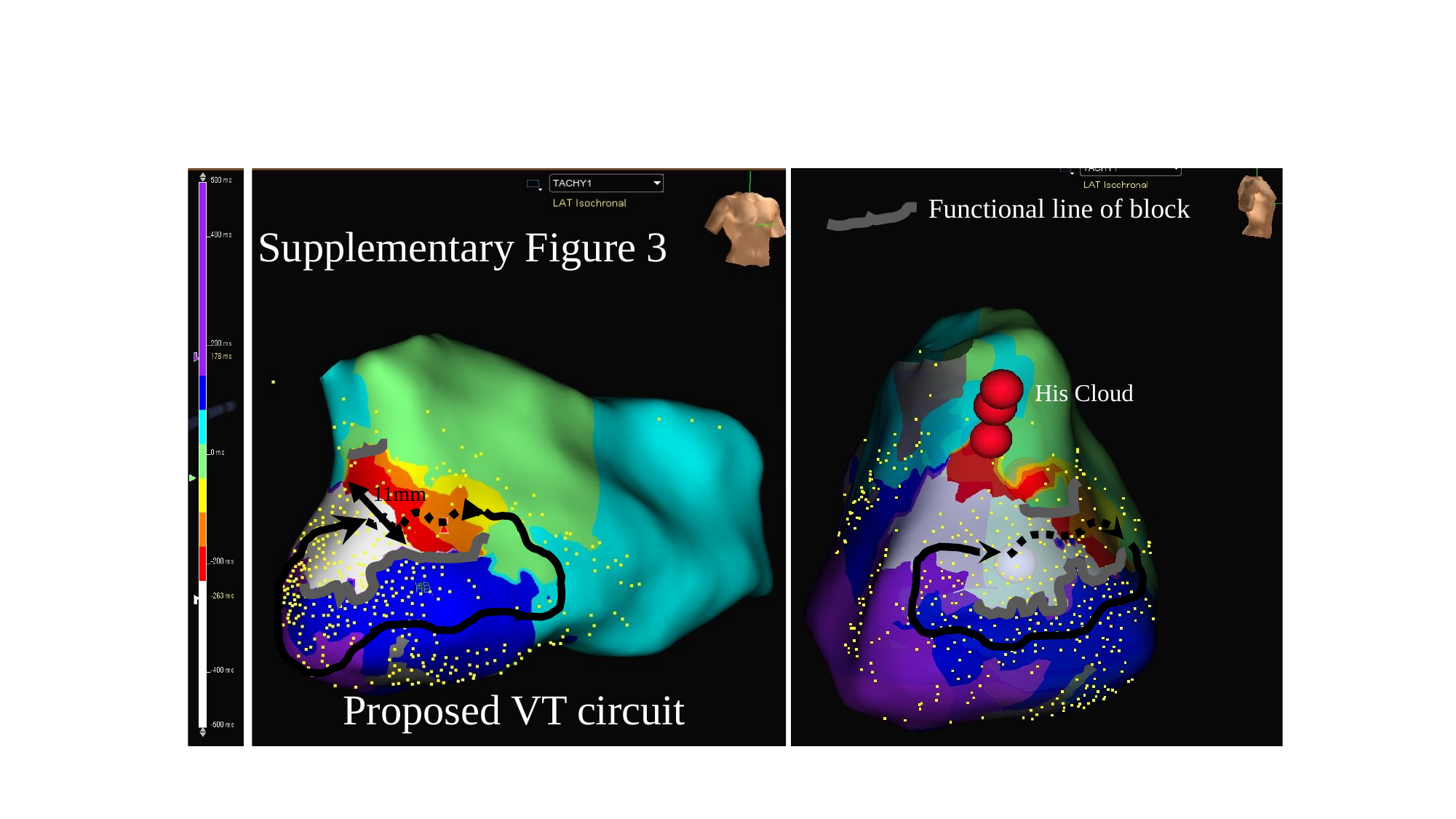

Supplementary Figure 3
Functional line of block
His Cloud
11mm
Proposed VT circuit

## Slide 4
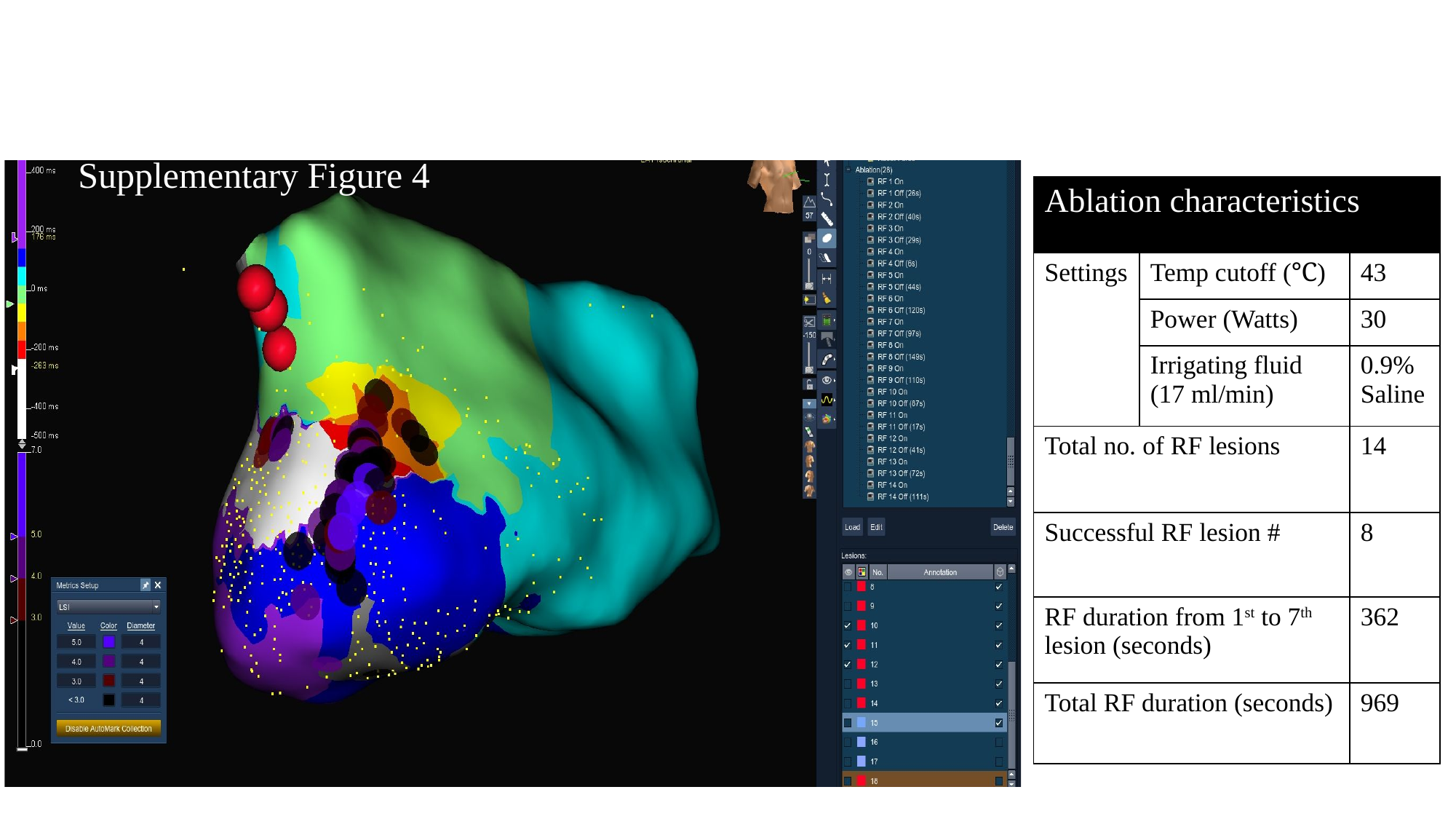

Supplementary Figure 4
| Ablation characteristics | | |
| --- | --- | --- |
| Settings | Temp cutoff (℃) | 43 |
| | Power (Watts) | 30 |
| | Irrigating fluid (17 ml/min) | 0.9% Saline |
| Total no. of RF lesions | | 14 |
| Successful RF lesion # | | 8 |
| RF duration from 1st to 7th lesion (seconds) | | 362 |
| Total RF duration (seconds) | | 969 |
